# SUBA4: the interactive data analysis centre for Arabidopsis subcellular protein locations

**DOI:** 10.1093/nar/gkw1041

**Published:** 2016-11-24

**Authors:** Cornelia M. Hooper, Ian R. Castleden, Sandra K. Tanz, Nader Aryamanesh, A. Harvey Millar

**Affiliations:** 1 ARC Centre of Excellence in Plant Energy Biology, The University of Western Australia, Perth, WA 6009, Australia; 2 Department of Genetics and Physiology, Biocenter Oulu, FIN-90014 University of Oulu, Finland

## Abstract

The SUBcellular location database for Arabidopsis proteins (SUBA4, http://suba.live) is a comprehensive collection of manually curated published data sets of large-scale subcellular proteomics, fluorescent protein visualization, protein-protein interaction (PPI) as well as subcellular targeting calls from 22 prediction programs. SUBA4 contains an additional 35 568 localizations totalling more than 60 000 experimental protein location claims as well as 37 new suborganellar localization categories. The experimental PPI data has been expanded to 26 327 PPI pairs including 856 PPI localizations from experimental fluorescent visualizations. The new SUBA4 user interface enables users to choose quickly from the filter categories: ‘subcellular location’, ‘protein properties’, ‘protein–protein interaction’ and ‘affiliations’ to build complex queries. This allows substantial expansion of search parameters into 80 annotation types comprising 1 150 204 new annotations to study metadata associated with subcellular localization. The ‘BLAST’ tab contains a sequence alignment tool to enable a sequence fragment from any species to find the closest match in Arabidopsis and retrieve data on subcellular location. Using the location consensus SUBAcon, the SUBA4 toolbox delivers three novel data services allowing interactive analysis of user data to provide relative compartmental protein abundances and proximity relationship analysis of PPI and coexpression partners from a submitted list of Arabidopsis gene identifiers.

## INTRODUCTION

The SUBcellular location database for Arabidopsis proteins (SUBA4, http://suba.live) has grown into a substantial collection of manually curated published data sets of large-scale subcellular proteomics, fluorescent protein visualization, protein-protein interaction (PPI), subcellular targeting calls from 22 prediction programs as well as a consensus algorithm.

**Figure 1.**
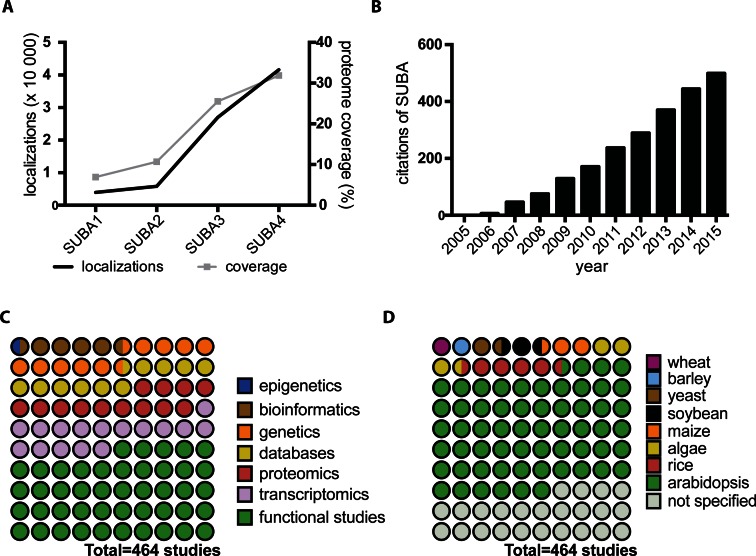
SUBA use in plant biology research. (**A**) The increase of collated localization data and coverage of the Arabidopsis proteome in SUBA releases from 2005 to 2016. (**B**) The accumulative citation record of the SUBA database based on SUBA1(4), SUBA2(5), SUBA3(6) and SUBAcon(7) reports between 2005 and 2015. Impact of SUBA data on (**C**) Plant biology research areas and (**D**) species-specific discoveries given as parts of retrieved studies that have cited SUBA.

The database originated from studies on the mitochondrial proteome >10 years ago (1). This mass spectrometry (MS) study of a single organelle revealed a large number of low-abundance proteins that had been predicted to localize elsewhere in the cell. Untangling which proteins were actually associated with mitochondrial functions and localized in mitochondria and which were low-level contaminants in purified mitochondrial preparation became a priority to resolve. Such analysis prompted the generation of the Arabidopsis Mitochondrial Protein Database (AMPDB) that offered an overview of the detailed MS data sets from 17 published mitochondrial studies as well as predictions from 6 subcellular location algorithms (2). Similar efforts for the plastid were realized as the Plastid Proteome DataBase (PPDB) at the same time (3). However, it was evident that each organelle should not be studied separately, and that the rapid expansion of data for many organelles required the establishment of a one-stop data collection for subcellular compartmentalization data. The initial data collation and categorization showed that data sets from different researchers had surprisingly little overlap and their combination provided an opportunity for large scale data mining (4). This report coined the SUBA acronym and revealed that protein families, subsets and isoforms with distinct subcellular location patterns existed. It also showed that experimental data was more variable and error-prone than the wider research community presumed. A description of the SUBA2 database in the Nucleic Acid Research Database issue followed in 2007 incorporating standardized TAIR6 genome annotations, GO-ontology, additional prediction algorithms and many more experimental data sets (5). Since then, the number of datasets and website functionality has been steadily increasing to incorporate new TAIR gene annotations, PPI data, more prediction algorithms (6), as well as high-confidence subsets (Arabidopsis SUbcellular REference—ASURE) and location consensus classifications (SUBAcon) (7), forming SUBA3 (6). The core experimental subcellular location data is now 10 times the volume in the original SUBA1. This represents an increased coverage of the proteome from 7 to 32% over the last decade (Figure 1A). Since the foundation of SUBA, Dr JL Heazlewood, Dr SK Tanz, Dr CM Hooper and Dr N Ayamanesh have been the key curators, while Dr J Tonti-Filippini and Dr IR Castleden have developed most of the GUI and database services to enable the user experience. A smaller fraction of manual subcellular curations were derived from TAIR (8), AmiGO and Swissprot.

With increasing data content, SUBA has gained recognition exceeding 500 citations averaging 30 published studies per year (Figure 1B). SUBA data has predominantly been used for elucidating functions of proteins or genes but also for improving data interpretation in the fields of transcriptomics, proteomics, genetics and bioinformatics (Figure 1C).

While SUBA has contributed mostly toward Arabidopsis research aiding the exploration of pressing plant biology questions, the data has also shown beneficial when researching agricultural hypotheses in rice, barley, maize, soybean and wheat (Figure 1D). The smaller fraction of crop research using SUBA highlights the importance of improving the linkage of SUBA across species-specific borders as well as the need to improve linkage of comprehensive subcellular data collections for non-model crop species (9,10).

For research, SUBA has contributed to the development of widely used organelle marker sets (11), protein family clone collections for functional genomics (12), as well as facilitated the functional elucidation of protein families involved in plant growth regulation (13). The latter resources and knowledge were used in over 900 downstream studies. More recently, SUBA has played a pivotal role in estimating plant cell energy budgets (14), exploring sugar metabolism networks in barley (15) and demonstrating sub-functionalization of gene family expansions (16). The breath of work benefitting from SUBA highlights the importance of ongoing efforts in developing this central subcellular resource.

With the upgrade to SUBA4, we have released additional new data types, collated more parameters, as well as novel interactive tools that enhance complex data interrogation by users in a straightforward way without the need of computational skills. The novel SUBA4 toolbox contains a number of tools that allow immediate access and analysis of the core SUBA data in linkage to three external data sources. This creates a new integrated SUBA data analysis centre that enriches SUBA data analysis options, discoverability as well as the re-use of published data derived from global experimental and computational efforts.

## MATERIALS AND METHODS

### Database structure and interface

SUBA4 data are stored in a MySQL relational database (MySQL 5.6), operating on a UNIX-based system (RHEL6) using Apache 2.2.15. Extra relational semantics were added using the Python 3.5 and SQLAlchemy library. The SUBA4 search portal is a web-browser based GUI (Graphical User Interface) written in dynamic HTML using Asynchronous JavaScript + XML (AJAX) to interact with the SUBA4 server.

The SUBA4 search portal can be used without prior knowledge of SQL and allows the construction of complex queries by selecting parameters in a point and click manner from the tabs in the search menu. The data can be accessed via http://SUBA.live and is compatible with most current browsers including Windows Explorer, Safari, Firefox and Chrome. The search window contains five query tabs that separate filter options into categories for ease of use. Each tab contains pre-formulated queries in full text with pull down menus and simple text boxes. The query window allows AND, OR and bracketing to create complex Boolean queries.

Query results can be accessed via the results tabs in a tabular format with a default row format containing protein identifiers and summaries of localization data. Each column contains a pull down menu for customizing the information shown. The results from a query may be downloaded as a csv tabular file for further analysis. Each protein hit in the results view is hyperlinked to a factsheet that provides further information including sequence, hydropathy profile, protein properties, annotations, coordinates of gene, coexpression or PPI partners and localization study description. Protein annotations are displayed as hyperlinks that provide rapid access to related resources on the same protein in UniProt, AmiGO, EMBL, Ensembl Plants/Gramene, KEGG, MSU and others.

### Arabidopsis proteins and annotations

SUBA4 data adds to the SUBA3 datasets and are based on the non-redundant Arabidopsis protein set obtained from The Arabidopsis Information Resources (TAIR release 10), and the Arabidopsis mitochondrial (117) and chloroplast (87) open reading frame (ORF) sets obtained from GenBank Y08501 and AP000423, respectively. SUBA4 contains 35 388 distinct protein sequences. The primary attributes of molecular weight, average hydropathicity and isoelectric point were described previously (6) and functional assignments (4) for each Arabidopsis locus were retained and updated as required for TAIR10. An additional 1,150,204 annotations (Supplementary Table S1) were integrated into SUBA4 for each AGI as a searchable format. The annotations were obtained from the data repository of Ensemble Plants release 31 (ftp://ftp.ensemblgenomes.org/pub/release-31/), Uniprot (http://www.uniprot.org/) and TAIR (https://www.arabidopsis.org/download/index.jsp). Individual databases were directly linked to the display on the SUBA4 output.

### Experimental localization data sets

Experimental subcellular localizations of proteins were sourced and collated using the methods previously described (6). In short, localizations by MS were obtained by searching PubMed (http://www.ncbi.nlm.nih.gov/pubmed) for the term ‘Arabidopsis AND (proteome OR proteomics) and by florescent protein tagging for the term ‘Arabidopsis AND (CFP OR GFP OR RFP OR YFP). AGIs, aliases and subcellular locations were extracted from text or supplementary data. In addition to the previously described 11 subcellular locations, 37 location subcategories were added, increasing the subcellular and suborganellar resolution of the data as a whole (Table 1).

**Table 1.** SUBA locations and SUBA4 expansion of subcompartmental locations

		
Major compartments (SUBA3)	Child location (SUBA4)	Grandchild locations (SUBA4)
cytoskeleton (GO:0005856)	intermediate filament (GO:0005882) microtubules (GO:0005884) actin filament (GO:0005884)	

cytosol (GO:0005829)	cell plate (GO:0009504) cytosolic ribosomes (GO:0022626)	

endoplasmic reticulum (GO:0005783)	ER lumen (GO:0005788) ER membrane (GO:0005789)	

extracellular (GO:0005576)	apoplast (GO:0048046) cell wall (GO:0009505)	

Golgi	Golgi apparatus (GO:0005794) trans-Golgi network (GO:0005802)	Golgi lumen (GO:0005796) Golgi membrane (GO:0000139) multivesicular body (GO:0005771) early endosome (GO:0005769)

mitochondrion (GO:0005739)	mitochondrial envelope (GO:0005740)	mitochondrial inner membrane (GO:0005743) mitochondrial outer membrane (GO:0005741)
	mitochondrial matrix (GO:0005759)	

nucleus (GO:0005634)	nuclear envelope (GO:0005635)	nuclear inner membrane (GO:0005637) nuclear outer membrane (GO:0005640)
	nuclear matrix (GO:0016363)	chromatin (GO:0000785) nucleolus (GO:0005730)

peroxisome (GO:0005777)	peroxisomal membrane (GO:0005778)	
	peroxisome matrix (GO:0005782)	

plasma membrane (GO:0005886)		

plastid (GO:0009536)	plastid envelope (GO:0009526) plastid stroma (GO:0009532) plastid thylakoid (GO:0031976)	plastid inner membrane (GO:0009528) plastid outer membrane (GO:0009527) plastoglobules (GO:0010287) plastid thylakoid lumen (GO:0031978) plastid thylakoid membrane (GO:0055035)

vacuole (GO:0000325)	vacuole membrane (GO:0009705) vacuole lumen (GO:0000330)	

The major compartments are hierarchically linked to subcompartments and enable downward retrieval or experimental localizations.

PPI subcellular localizations were obtained by searching PubMed (http://www.ncbi.nlm.nih.gov/pubmed) for the search term ‘Arabidopsis AND (interact OR interaction OR protein–protein)’. The retrieved literature was manually searched for BiFC and co-localizations. PPI experimentation and subcellular locations were extracted from this literature into the 49 categories (48 locations + "unclear"). SUBA4 contains 122 PPI localization studies, collating 683 pairs observed in 858 localizations covering 29 of location categories. Overall, Arabidopsis PPI datasets of 16 252 protein pairs were obtained from the IntAct database (http://www.ebi.ac.uk/intact/) and 8990 PPI protein pairs were manually curated as previously described (6). In total, 21 495 unique protein pairs were integrated in SUBA comprising 26 327 experimental individual verifications.

Arabidopsis gene coexpression pairs, mutual rank and average correlation coefficient data were sourced from the ATTED-II database (http://atted.jp) as described (7,17). Coexpression between identical AGIs (self) were omitted from the list.

The subcellular targeting predictions for 22 bioinformatic programs were described in SUBA3 (6). The SUBAcon consensus was retrained as previously described (7) using updated experimental data and the updated Arabidopsis SUbcellular REference standard (ASURE).

### SUBA4 tools

The BLAST, Coexpression Adjacency Tool (CAT), PPI Adjacency Tool (PAT) and Multiple Marker Abundance Profiling (MMAP) tool were developed to aid the SUBA4 user. The BLAST tool was build using a standard BLASTX method (18). The score shown is −log_2_(*E*) where *E* = pval × Neff is the *P*-value times the effective search space size. The score increases with sequence match. Users can enter full or parts of protein sequences, where protein sequences with gaps are treated as separate fragments that are matched against Arabidopsis proteins independently. For the CAT and PAT tools, the coexpression data and PPI data sets were linked to SUBAcon calls. The SUBAcon calls of each AGI partner was joined and categorized into proximity relationships according to their biological interpretation (Supplementary Table S2). The subcellular locations, proximity relationships, mutual rank and average correlation coefficient data were linked to the tool interface as a searchable parameter.

The MMAP tool will be described in detail elsewhere (personal communications, H.T. Parsons *et al*.). In short, spectral counts collated as described previously (19,20) were sourced from the MASCP gator (http://www.masc-proteomics.org) for each AGI. The spectral counts of all experimentation were normalized using the Normalized Spectral Abundance Factor (NSAF) method (21). Before linking to the SUBAcon output, the NSAF values were normalized and scaled to the total sum to generate Gator scores. Using a marker list of >17 000 high confidence classification proteins, their Gator score and location call, calculations were performed to estimate the organellar protein abundance as a relative fraction of overall expected protein abundance in a cell. The distribution of organellar proteins (SUBAcon) and organellar protein abundance (MMAP) are displayed as easy-to-interpret bar and pie charts.

## UPGRADE IMPLEMENTATION

### Expanded datasets and annotations

#### Increased localization resolution for experimental data

Historically, SUBA location categories adhered to 11 locations seen as major compartments or organelles in plant cells (Table 1). This has been a practical starting point for consolidating the initial data sets that relied on different types of experimental data with varying resolution. Since then, the technological advancements in microscopy and organellar extraction has led to increasing production of large scale data for more cellular subcompartments (22–24) as well as high resolution data for plastid subcompartments such as thylakoid membranes (25) and plastoglobules (26). Also, high-resolution fluorescence protein (FP) (27) as well as fluorescence-based PPI localization data (28) now exist with greater ability to locate proteins more precisely. To enable the use of these data in SUBA4, 37 additional subcellular categories were introduced (Table 1) bringing the total number of location categories for experimental data to 48. The categories were determined based on available data, biological relevance and ongoing curation efforts (29). Experimental data can be filtered for these locations through the SUBA4 interface. In order to maximize query returns, localization data are aligned to GO-ontology and structured in hierarchical order to ensure users receive all data matching the query location as well as associated child locations.

**Figure 2.**
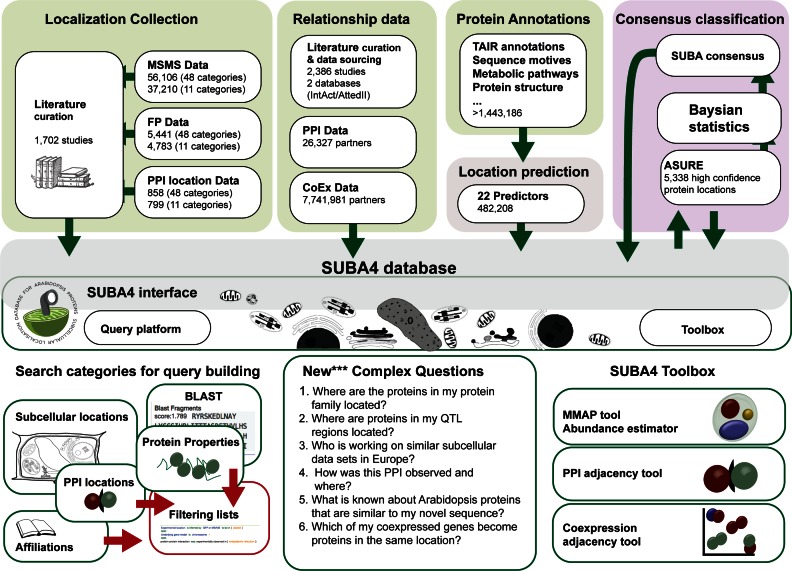
SUBA4 curation, calculations, predictions, classifications data and interrogation overview. The SUBA4 data collation (top) is accessible through the interface (middle). The interface offers the query platform (bottom left) for filtering data and the toolbox (bottom right) for rapid statistics. *ASURE—Arabidopsis SUbcellular REference, CoEx—coexpression, FP—fluorescent protein, MS—mass spectrometry, PPI—protein–protein interaction*.

Using the new location categories, both newly collated as well as previously collated localization data in SUBA were re-built to create the SUBA4 experimental data localization sets for MS, FP and PPI data. Comparing to the SUBA3 release in 2013, SUBA4 contains 37 210 (from 22 191) MS, 4783 (from 3788) FP and 799 PPI (new data source) localizations mapped to the original 11 categories. Using the increased localization categories or previously and newly collated reports of protein locations, this increases to 56 106 MS, 5441 FP and 858 PPI localizations covering 11,231 distinct proteins, ∼32% of the Arabidopsis proteome in total (Figure 2).

#### Expanded parameter and localization searchability for PPI data

As part of the SUBA3 curation process, PPI data not available through the IntAct platform was manually curated and collated. We have now provided the collated localization data of fluorescent methodology-based PPI experimentation in these studies. The complete SUBA4 PPI collection contains 26 327 individual PPI observations of 21 495 distinct protein pairs. Of these, 858 experimentations can be searched for their subcellular and suborganellar locations. This feature allows the user to investigate specific proximity environments when assessing PPI relationships. We aligned our PPI methodology data to the categories of IntAct, which allows the users to filter for specific PPI methodologies including yeast-2-hybrid (Y2H), bifocal completion (BiFC), pull-down, co-localization, co-immunoprecipitation, Luciferase, fluorescence resonance energy transfer (FRET) and fluorescence lifetime imaging (FLIM)-FRET and a category ‘other’ combining a number of alternative methodologies across both the IntAct and manually collated data. The option to filter for PPI methodologies allows the user to control some of the well-known biases derived from specific methodologies (30) for downstream data analysis.

#### Metadata and annotation upgrade

With improving metadata standards within electronic libraries, we have expanded the options to explore metadata affiliated with the experimental studies. SUBA4 now offers filter options for PMIDs, year range of publications but also allows geographical filters for data by institution and countries. A large number of newly available protein annotations were obtained from Gramene (31), TAIR (8) and Uniprot (32) browsers that after collation amounted to 1 443 186 annotations from 80 different data types (Supplementary Table S1). All annotations have been introduced as searchable entities allowing filtering for structure, pathway associations and other protein or gene properties. The additional features allow novel connections and exclusions of previously separate data and metadata sets.

### A categorized search interface for quick access to varied filter options

**Figure 3.**
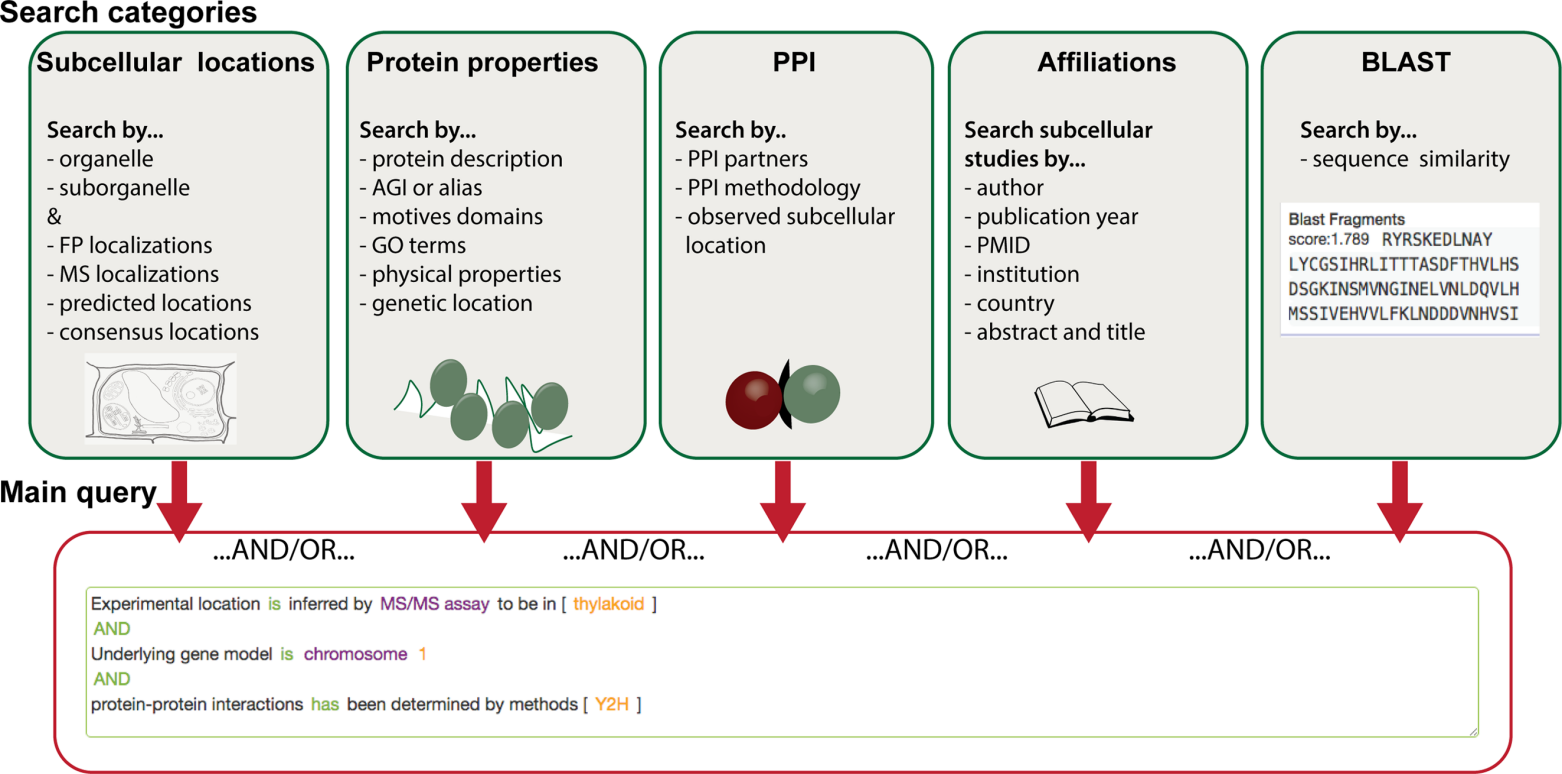
SUBA4 query platform overview. Each query category is summarized in a green box (top) indicating individual filter options. The filters from each category can be linked into one main query using Boolean AND/OR terms as indicated in the red box (bottom). *FP—fluorescent protein, MS—mass spectrometry, PPI—protein–protein interaction*.

The integration of new parameters, data sets and annotations allows more query options that link distinct data modalities. In order to rapidly find and assemble user-specific research questions through the SUBA portal, the search interface was upgraded to a tab-category system (Supplementary Figure S1). The tab-category system separates the query building blocks in intuitive sections for subcellular, protein properties, PPI, affiliations and BLAST questions (Figure 3). The subcellular tab shows filter options for experimental MS and FP data or for predictive subcellular locations by the 22 predictors and the SUBAcon classification. The newly integrated suborganellar categories for experimental data can be accessed by clicking on the arrow ‘»’ for each organelle. Any of these can be chosen as a search filter. For ease of use, the cell schematic for the 11 major locations is provided for users to click on the desired organelle. In SUBA4, more than one organelle can be selected at the same time, before adding the filter to the main query. The locations will be connected by an ‘OR’.

The protein properties tab contains all filters regarding physical protein properties, genetic locations as well as all AGIs, TAIR descriptions, and all other searchable annotations (Supplementary Figure S2). The latter allows users to retrieve whole protein families or groups associated with specific GO, PFAM terms and other annotations and assess their subcellular locations.

In order to manage complex queries specifically associated with the PPI data, a PPI tab was generated (Figure 3). Beside the conventional queries identifying existing PPI partners, users can now also filter for methodology and observed PPI subcellular location. The subcellular location search layout for PPI data is the same as for MS and FP. PPI localizations are derived from manually curated bifocal completion and fluorescence resonance energy transport assays in studies and constitute an additional localization data set to existing FP and MS data.

Newly available bibliography metadata from research studies can now be searched through the affiliations tab (Figure 3). It contains a large number of filter options that can be combined to a specific query about the age of data and the research groups responsible. The world map can be used to quickly assess which countries have contributed data sets to SUBA4 (Supplementary Figure S3) and the full detail of the global distribution of the studies can be seen under the Locations tab in the top menu.

The last tab contains a BLAST sequence alignment tool new to SUBA4 (Figure 3). The BLAST tool is linked to the SUBA search function to enable users with sequence fragments from all species to find the closest match in TAIR10 and directly retrieve data from SUBA. The alignment and BLAST score of the submitted sequence to a matching AGI in SUBA are shown in the results view for each hit. This extends the SUBA data user base and aligns with the general global efforts of connecting species data more easily (33). A quick link window to the BLAST tool can also be found directly on the SUBA4 homepage.

The query-building window is connected to all five tabs (Figure 3), allowing the user to choose filters from any tabs to build one query. The new SUBA4 tab system allows for scalability to future parameters and added functionality without the need of interface changes.

### A toolbox to view and interrogate large-scale subcellular location data

The toolbox in SUBA4 contains three distinct tools, namely the Multiple Marker Abundance Profiling (MMAP) tool, Coexpression Adjacency Tool (CAT) and PPI Adjacency Tool (PAT). Each tool provides a unique link between the subcellular location consensus (SUBAcon) and protein abundance (MMAP), coexpression (CAT) and PPI (PAT) data, respectively, giving the SUBA4 user straightforward data analysis options. The detailed descriptions of the development of the MMAP, CAT and PAT and an assessment of their uses will be described in detail elsewhere (personal communications, H.T. Parsons *et al*.). However, a brief introduction to each tool is given here.

**Figure 4.**
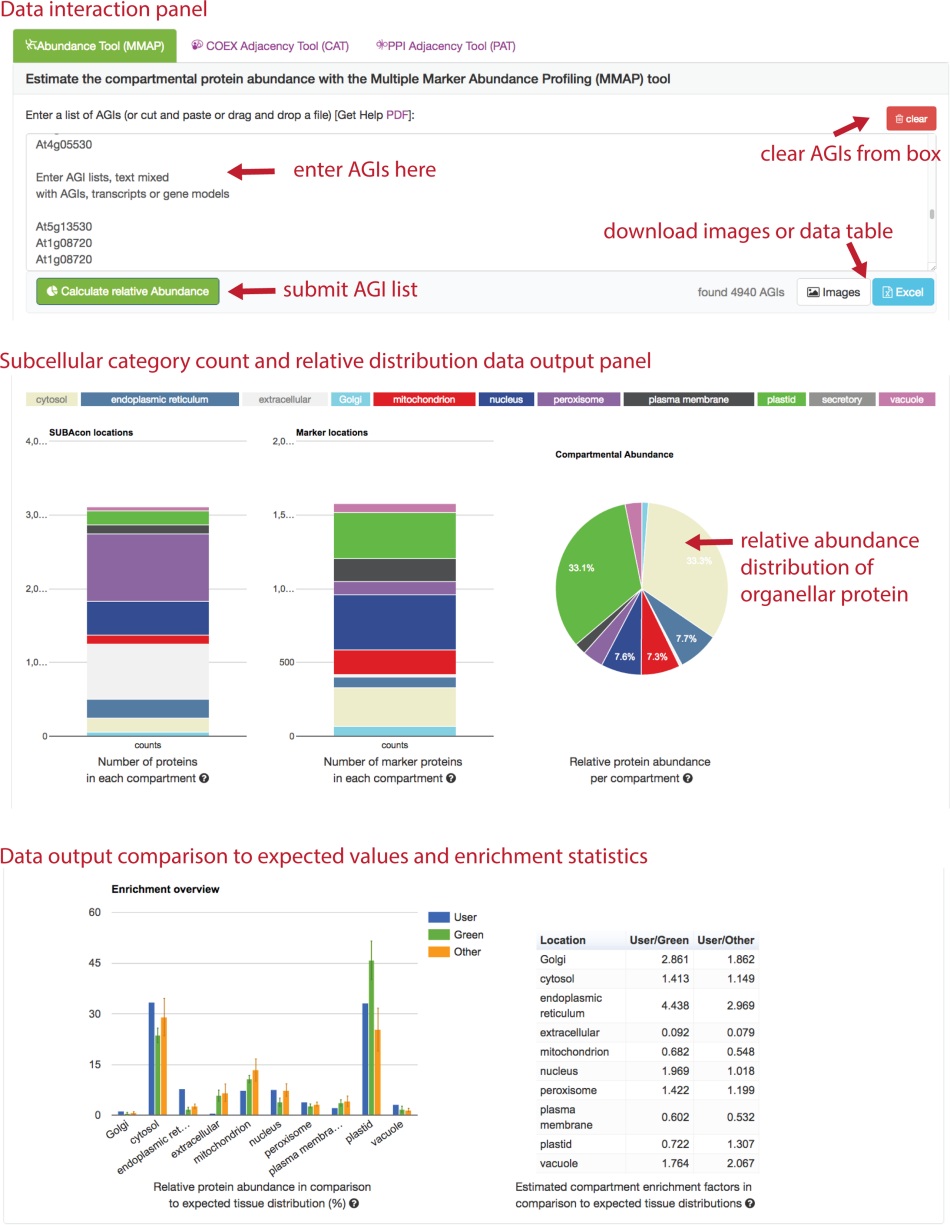
SUBA4 MMAP tool overview. The MMAP tool in the toolbox tab accepts lists or text containing AGIs in the top window (top panel). The MMAP tool returns summarized counts of protein species per compartment as well as the relative protein abundance estimation per compartment (middle panel). The bottom panel returns an enrichment factor relative to the expected compartmental protein abundance distribution in green or other tissues. *MMAP—Multiple Marker Abundance Profiling*.

The MMAP tool provides an estimate of the proportion of different subcellular protein structures in a user provided list of AGIs by combining information from SUBA and MS experimental observations of proteins in the MASCP gator database. All spectral counts observed in MASCP gator were scaled to estimate a relative abundance score of each protein as a measure of observed spectral counts across all experiments. A marker list of single targeted proteins with high confidence was generated that in combination with the observation abundance score was used to estimate the relative abundance of organellar protein in a given AGI list. Therefore, users can submit custom AGI lists to the MMAP tool and receive the number of distinct proteins per each organelle as well as an estimate of relative protein abundance composition compared to expected subcellular abundances in green (high-light) or other (low-light) tissues (Figure 4).

**Figure 5.**
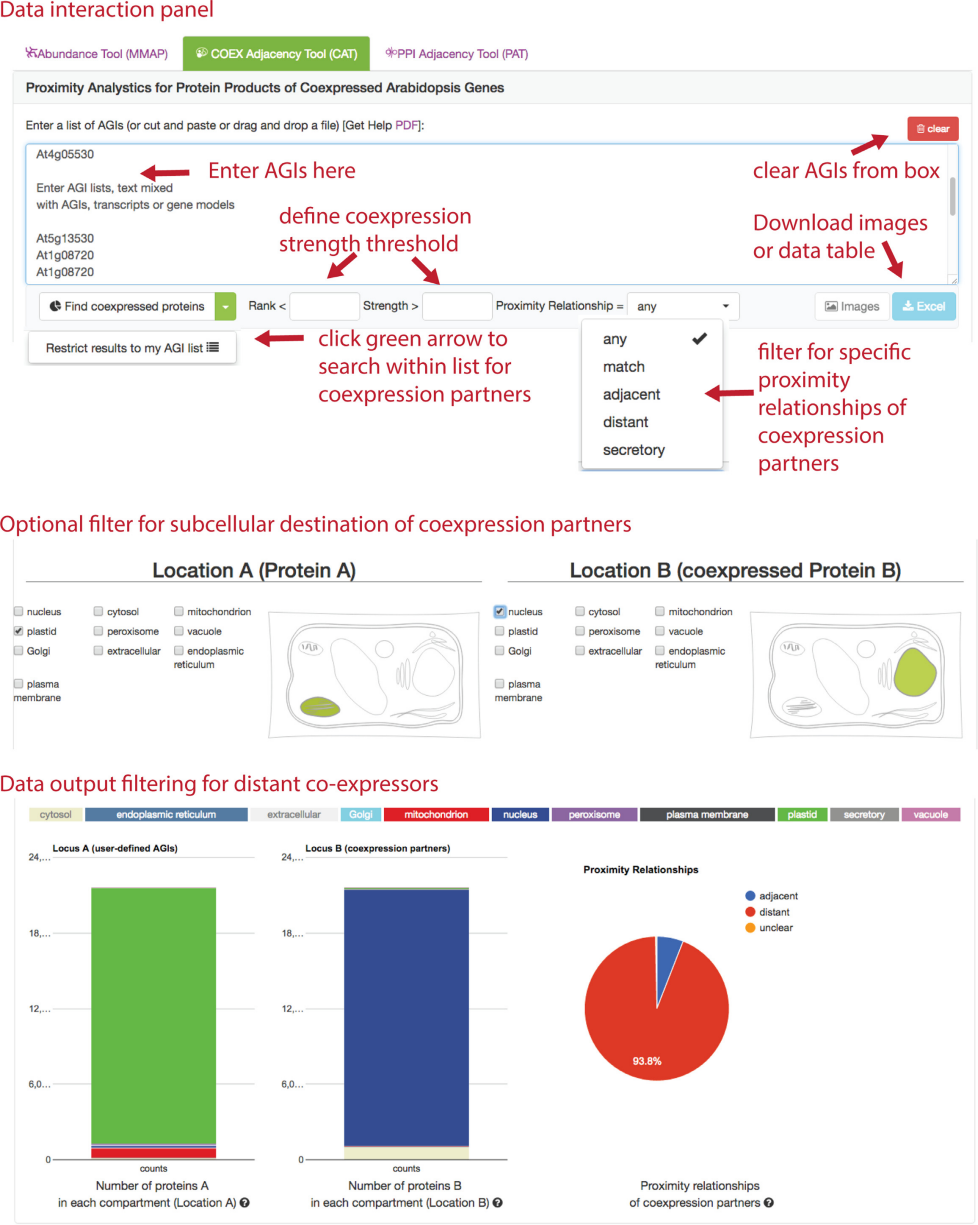
SUBA4 Coexpression Adjacency Tool (CAT) overview. The CAT accepts lists or text containing AGIs in the top and allows filter options for coexpression strength and proximity relationships of proteins derived from coexpressed genes (top panel). The submit button allows to ‘find coexpressed proteins’ for all AGIs or to narrow coexpression partners to within the submitted AGI list. More filter options in CAT allow specifying subcellular locations of AGIs in the submitted list as well as the returned coexpression partners (middle panel). The tool returns a protein type distribution for proteinA and proteinB as well as the distribution of proximity relationships (match, adjacent, distant, secretory) within a submitted list or associated with a submitted list (bottom panel). The distributions are returned as relative bar charts and pie charts.

The CAT tool allows the coexpression of genes encoding specific proteins in a list to be assessed in context of the final subcellular location of their proteins. It makes use of the fact that when two transcripts are coexpressed there is a potential relationship between the proteins they encode. The user can assess a list of AGIs for existing coexpression partners that are both within the list or with only one AGI within the list. The search can be filtered by coexpression strength parameters - rank and correlation coefficient (Figure 5). The tool offers filter options for subcellular locations of ProteinA and coexpressed ProteinB or allows the retrieval of protein partners with the proximity relationship categories: match, adjacent, distant or secretory. This proximity relationship classification was developed using the paired call of the SUBAcon classification of both proteins (Supplementary Table S2). After submitting the list of AGIs, the tool uses the SUBAcon calls, collates them and compares them to the key, the proportions of the proximity relationships in the queried data set is then displayed in the results offering an instant analysis of the coexpression data in a subcellular context (Figure 5).

The PAT tool is built on similar statistical principles and relationship categories to the CAT tool but using experimental PPI data. SUBA4 contains 26 327 unique experimental PPI observations, of which only 799 have been experimentally localized during the PPI experiment. Using SUBAcon, all proteins from the PPI data set can be assigned to a subcellular location, that when paired indicate a proximity relationship of the PPI couple. The PAT tool was developed to enable interpretation of protein-protein relationships in the context of proteins proximity in the cell. The user can identify a number of experimentally verified PPI partners within a list or associated with a protein in a submitted AGI list through the PAT tool. The user can also take advantage of filter options regarding the classified subcellular location of proteinA and proteinB or filter by the proximity relationship of the protein pairs (Supplementary Figure S4).

The CAT and PAT tools will aid the interpretation of typical subsets of AGIs derived from clustering, filtering of transcript data including sets of proteins changing in abundance in particular conditions or genotypes, protein lists from immunoprecipitations, organellar or membrane preparations, lists of proteins in pathways or gene families and many other custom questions posed by the user.

### A simplified results view with more customizable options

When submitting a query, the results tab automatically becomes activated and shows the hits in tabular format. Since SUBA3, the default content presentation of the retrieved SUBA data (AGI, protein description, SUBAcon location calls, predictions and experimental data) has been simplified to avoid overloading the viewing field. The user can choose from a number of additional display options including locations predicted, external curation, experimental data, PPI, and bibliography metadata. The experimental data in the SUBA4 results table is summarized per organelle indicating the amount of experimental data available from FP and MS. The last column displays the PPI data including the AGI and now the SUBAcon call for any interacting proteins. The simplification and added flexibility for data display in the results table caters for users with distinct questions. A user can now revisit the query that led to the results by clicking on the ‘What's this Query?’ button. The ‘Download’ button next to it will initiate the download of the query result data table for use of SUBA data for downstream applications. For detailed investigation of individual proteins, the user can click on the AGI in the results view and open the individual AGI factsheet.

### A factsheet that visually collates subcellular and annotation data

The additional suborganellar data, PPI parameters, annotations and data set linkage substantially increased the amount of data for each AGI available. The SUBA4 factsheet has been redesigned to separate prediction from experimental localizations as well as include protein-protein proximity relationships (Supplementary Figure S5). The factsheet, similar to SUBA3 provides a data summary (SUBAcon, ASURE and schematic), predictions and external curations at the top. The increased depth of subcellular location is shown for FP, MSMS and PPI studies as a column, while the PMID, year, AGI of PPI partner PPI method and observed location (if available) is visible for PPI localizations associated with each AGI. A further category of protein-protein relationships was added that links SUBAcon calls directly to coexpression data as well as all PPI data. Thereafter, associated AGIs by coexpression (rank 1–10) and PPI are collected and linked to individual SUBAcon calls. The output gives the viewer an immediate overview of affiliated gene networks and PPI links and their subcellular locations. If the overview on the factsheet reveals a lead, more detailed investigations can be performed using the CAT and PAT in the toolbox. Finally, while retaining the conventional parameters of the SUBA3 factsheet, SUBA4 also contains all newly incorporated annotations that link directly to the respective data source for further exploration.

### Current implementation status and future plans

SUBA provides a landing portal for accessing and linking existing subcellular data sets fast, which increases visibility and reuse of existing valuable experimental data. SUBA has become a central go-to for subcellular information in plant biology over the past decade. In context of ongoing global data maintenance efforts, SUBA has evolved alongside guidelines of data structuring, provenance and standardization consensus agreements around TAIR and Ensembl Plants.

SUBA4 now highlights the value of data linkage between subcellular data and other data entities from transcriptomics (AttedII) and proteomics (MASCP gator) in the SUBA4 toolbox. In reverse, many omics analysis pipelines benefit from SUBA-derived subcellular annotations. Currently, SUBA is integrated in the Bio-Analytic Resource platform (http://bar.utoronto.ca/) aiding transcriptome data interpretation as well as in Araport (https://www.araport.org/) as an intergrated API for downstream analysis pipelines. Further efforts aim to link SUBA to global genome and proteome browsers such as Gramene and Uniprot, respectively.

Future plans see SUBA utilize subcellular-refined pathway mapping to predict and define subcellular reaction rooms in plant cells. More than 35 000 Arabidopsis proteins are predicted to exist of which only 10% have been used in genome-wide metabolic models for calculating the energy household of plant cells (34–37). In fact, all of these models have heavily relied on SUBA to generate the compartmentalization of metabolism, which in turn achieved a better representation for estimating the energy required for cell homeostasis (14). With the steadily growing subcellular resolution, SUBA will remain an important part of building increasingly compartmentalized and complete genome-wide metabolic models. SUBA4 has now directly linked several pathway-mapping services including KEGG and Biocyc to the subcellular location data. Together with pathway prediction tools and subcellular location, the SUBA toolbox plans to provide useful cataloguing pipelines that help predict pathways, pathway linkage across compartments as well as necessary transporters into balanceable reaction rooms for modelling bioprocesses more accurately. Similarly, new quick tools for linking SUBA with other data entities such as phenomics data and epigenetic data will be an important step toward linking molecular data to plant traits. The latter data sets, while currently challenging to link, are under development for implementation into the SUBA toolbox for future SUBA releases.

## CONCLUSIONS

We have developed a substantially upgraded interface and new data sets through the SUBA4 website at http://SUBA.live. The upgrade reflects the increased resolution of biological data as well as the challenge to provide links and searchability across multimodal data types to yield meaningful insights into biological processes. The collection of annotations and protein/gene properties enables systematic interpretation of the subcellular data in Arabidopsis from multiple viewpoints. With the SUBA4 toolbox we have delivered a quick and straight-forward data service that allows the user to statistically analyse subcellular location data linked to protein abundance, coexpression and PPI for a defined list of proteins. As many plant scientists handle large data lists, these tools will accelerate data analysis that leads to new hypothesis formation by increased data linkage. In addition, the direct BLAST-to-SUBA function allows assessment of new sequences from other plants by SUBA and increases options for cross-species analysis.

## AVAILABILITY

The SUBA4 data set is available for bulk download through the Research Data Online repository (http://www.library.uwa.edu.au/research/rdo) under DOI: http://dx.doi.org/10.4225/23/581055ddcb1ce.
